# Virtual brain tumours (gliomas) enhance the reality of medical imaging and highlight inadequacies of current therapy

**DOI:** 10.1038/sj.bjc.6600021

**Published:** 2002-01-07

**Authors:** K R Swanson, E C Alvord, J D Murray

**Affiliations:** Department of Applied Mathematics, Box 352420, University of Washington, Seattle, Washington WA 98195, USA; Department of Pathology, Box 359791, University of Washington, Seattle, Washington WA 98104, USA; Laboratory of Neuropathology, Harborview Medical Center, 325 – 9th Avenue, Seattle, Washington WA 98104-2499, USA

**Keywords:** brain tumour, glioma, mathematical model, theoretical model

## Abstract

Gliomas are brain tumours that differ from most other cancers by their diffuse invasion of the surrounding normal tissue and their notorious recurrence following all forms of therapy. We have developed a mathematical model to quantify the spatio-temporal growth and invasion of gliomas in three dimensions throughout a virtual human brain. The model quantifies the extent of tumorous invasion of individual gliomas in three-dimensions to a degree beyond the limits of present medical imaging, including even microscopy, and makes clear why current therapies based on existing imaging techniques are inadequate and cannot be otherwise without other methods for detecting tumour cells in the brain. The model's estimate of the extent of tumourous invasion beyond that defined by standard medical imaging can be useful in more accurately planning therapy regimes as well as predicting sites of potential recurrence without waiting for reemergence on follow-up imaging.

*British Journal of Cancer* (2002) **86**, 14–18. DOI: 10.1038/sj/bjc/6600021 www.bjcancer.com

© 2002 The Cancer Research Campaign

## 

Gliomas are brain tumours that differ from most other tumours by their aggressive diffuse invasion of the surrounding normal tissue. This invasive nature contributes to their dismal 6 to 12 month prognosis (Silbergeld *et al*, 1991). The remarkable continuing development of medical imaging has increased the detection of gliomas but has not been able to sufficiently define the degree of diffuse invasion of the tumour cells peripheral to the bulk of the tumour mass to allow for adequate assessment and treatment. Thus, it is not surprising that even extensive surgical resection or local irradiation of gliomas is followed by tumourous recurrence at or near the edge of the resection bed (Gaspar *et al*, 1992; Liang and Weil, 1998).

Mathematical modelling of biomedical phenomena (Murray, 1993) can be extremely helpful in analyzing factors that may contribute to the complexity intrinsic in insufficiently understood developmental processes and diseases. Despite the complexity of gliomas some of the basic components of this disease have been illuminated. Based on present knowledge of the properties of gliomas, we have developed a mathematical model to quantify the spatio-temporal proliferation and invasion dynamics of gliomas within anatomically accurate heterogeneous brain tissue in three spatial dimensions. The implications of this type of modelling would be of considerable interest to not only neuro-oncologists attempting to improve the treatment of gliomas but also to those interested in the quantitative study of other diseases for which medical imaging plays a large part of the assessment of the disease (e.g. other cancers as well as developmental diseases).

There are, of course, many properties of gliomas, but we believe that these can be grouped together yeilding two key parameters: net proliferation rate and migration rate of cells. Previous models based on these assumptions have studied the interaction of these two characteristics of gliomas with considerable success (Cruywagen *et al*, 1995; Tracqui *et al*, 1995; Woodward *et al*, 1996; Burgess *et al*, 1997). These previous mathematical models of gliomas considered the brain to be a two-dimensional homogeneous tissue bounded only by the ventricles and skull (dura or arachnoid) (Cruywagen *et al*, 1995; Tracqui *et al*, 1995; Woodward *et al*, 1996; Burgess *et al*, 1997). Even with this anatomic simplicity, the models could predict the behaviour of untreated high-, intermediate- or low-grade gliomas (defined by differences in the motility and proliferation rates) (Woodward *et al*, 1996). Even more interestingly, the previous models could predict the behaviour of surgically treated high-grade gliomas to a degree of accuracy not attainable *in vivo* with ‘statistically significant probability’ even with groups of over 50 real patients (Kreth *et al*, 1993).

Our interest is in the transfer of these predictions from groups of virtual patients to individual patient cases. To facilitate this, it was necessary for our model to reflect both the gross and microscopic anatomic complexity of the human brain. The availability of the BrainWeb (Collins *et al*, 1998) brain atlas database (http://www.bic.mni.mcgill.ca/brainweb) has allowed us to refine the gross anatomic boundaries of the human brain in three-dimensions. By defining a virtual human brain with the anatomical distribution of grey and white matter (the two primary tissue components of the brain) to a voxel resolution of 1 mm^3^, we can model the differential motility of glioma cells in grey and white matter to accommodate reports (Giese *et al*, 1996) that such differences are biologically significant. Specifically, since glioma cells are reported to migrate more rapidly in white matter than in grey matter (Giese *et al*, 1996; Silbergeld and Chicoine, 1997; Swanson, 1999) we allow the motility coefficient to differ depending on the local tissue composition.

## MATERIALS AND METHODS

### The mathematical model

Our mathematical model for glioma growth and invasion quantifying the differential motility of gliomas in grey and white matter can be written, in words, as:

the rate of change of the tumour cell population density =
the diffusion (motility) of the tumour cells in grey and white matter + the net proliferation of the tumour cells

and can be quantified mathematically as:


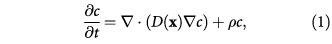


where *c(**x**,t)* is the concentration of tumour cells at location **x** and time *t*. *D(**x**),* a function of position ***x*** in the brain, is the diffusion coefficient defining the random motility of the glioma cells with *D(**x**)=D_g_, D_w_*, constants for **x** in grey and white matter, respectively. ρ represents the net proliferation rate of the glioma cells and ▿ is the spatial differentiation operator (in effect a gradient). As is observed biologically, the diffusion (motility) coefficient in white matter is larger than that in grey, that is, *D_w_* >*D*_*g*_. The difference in the diffusion coefficients in grey and white matter has been estimated to range from 2–100-fold (Swanson, 1999), but we have chosen 5 as an arbitrary first approximation to illustrate the model's potential. To complete the model formulation, we impose a ‘zero-flux’ of cells across the brain boundaries defined by cerebrospinal fluid in the ventricular and subarachnoid space. This boundary condition simply requires that glioma cells are not allowed to migrate outside of the brain tissue. We also assume that the tumour has grown to about 4000 cells as a local mass before it begins to diffuse (and the model equation (1) applies) in order to avoid simulating a case of gliomatosis cerebri for which tumour cells have invaded throughout the brain without a single dominant tumour mass.

### Definition of anatomic landmarks in 3-dimensions

Previous models (Cruywagen *et al*, 1995; Tracqui *et al*, 1995; Woodward *et al*, 1996; Burgess *et al*, 1997) considered the brain to be homogeneous, bounded only externally by the skull and internally by the ventricles. With the BrainWeb atlas we have been able not only to refine the external boundaries to the complex sulci (hills and valleys of the human brain cortex) to a voxel resolution of 1 mm^3^, but also to allow the virtual glioma to grow and migrate within a true 3-dimensional representation of the human brain allowing each 1 mm thick slice of brain to interact with grey and white matter in adjacent planes. As for the spread of gliomas across sulci we have no specific information as to how difficult it is for glioma cells to cross that barrier. We know that it can occur *in vivo*. We suspect that it is considerably more resistant than the boundary between grey and white matter (where we have assumed a factor of 5 difference in motility). We have assumed, for the purpose of the present demonstrations, that the sulci (pial) barrier is absolute, but this is extreme and could easily be revised as more information becomes available.

### Parameter estimation

Essential to successful modelling is the ability to determine reasonable estimates of the critical parameters, the growth rate ρ and the average diffusion coefficient *D* (an average of the diffusion coefficients in white matter *D_w_* and grey matter *D_g_*). For high-grade gliomas (glioblastomas) previous reports, based on extant data, have suggested a net proliferation rate of ρ≈0.012/day (Alvord and Shaw, 1991; Swanson, 1999; Swanson *et al*, 2000), corresponding to a volume-doubling time of 60 days, and a diffusion coefficient of *D*≈0.0013 cm^2^ day^−1^ (Swanson, 1999; Swanson *et al*, 2000). The actual ranges of these values are quite extreme but real values for any actual patient could be substituted. Variation of the proliferation rate and diffusion coefficient by 50% has been previously shown to reproduce the survival curves observed for high-grade gliomas (Woodward *et al*, 1996). We discuss elsewhere means of calculating these parameter estimates by the use of standard imaging data for individual patients (Swanson *et al*, submitted). To calculate the parameters ρ and *D*, we look to the medical images necessary for the diagnosis of gliomas.

### Thresholds of detection

For every medical imaging technique, be it CT, MRI, gross or microscopic studies, there is a threshold of detection below which glioma cells are not detectable. Clearly, CT and MRI have a higher capability for detection of gliomas than gross examination of an autopsy specimen but even microscopy has a limit beyond which individual cells cannot be detected. For example, close to the extreme, imagine a low concentration of tumour cells of the order of 1 cell in a pin-head mass of tissue, about 1 mm^3^. Simple arithmetic calculations show that at a concentration of 1 cell mm^−3^ it would require 100 serial sections each 10 μm thick to detect this single cell, assuming that the cell could even be recognized by any specific histologic technique. Confirmation of this analogy has been provided by Silbergeld and Chicoine (1997), who were able to grow in culture glioma cells from brain tissue that was histologically normal 4 cm distal to the gross edge of the tumour. Furthermore, Kelly *et al*. (1987) were able to show in at least half of their patients that ‘isolated tumour cells’ could be identified microscopically in biopsies distal to even the most sensitive MRI marker.

Previous reports (Burgess *et al*, 1997) estimated the enhanced CT-delineated tumour boundary to correspond to a tumour cell concentration of about 8000 cells mm^−3^. We retain this estimate, which corresponds to about 400 cells in a low power (10× objective) microscopic field of about 1 mm^2^ or about 25 cells in a high power (40× objective) field.

### Survival time

Previous models assumed that diagnosis is made when the volume of an enhanced CT-detectable tumour has reached a size equivalent to a sphere with an average 3 cm diameter and that death occurs when the volume reaches an average 6 cm diameter. The difference between these two times can be defined as the survival time of the hypothetical or virtual patient. With earlier even simpler models, the comparison of calculated survival times (Woodward *et al*, 1996) with extant data (Kreth *et al*, 1993) was very good.

## RESULTS

### Virtual gliomas

Figure 1

**Figure 1:**
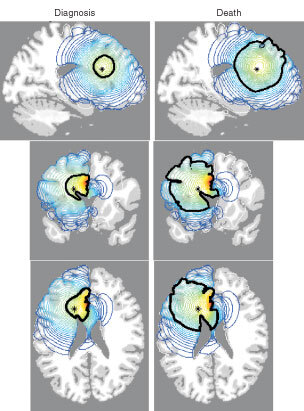
Sections of the virtual human brain in sagittal, coronal and horizontal planes that intersect at the site of the glioma originating in the superior frontal region denoted by an asterisk (*). The left column of brain sections corresponds to the tumour at diagnosis whereas the right column represents the same tumour at death. Red denotes a high density of tumour cells while blue denotes a low density. A thick black contour defines the edge of the tumour detectable by enhanced CT. Cell migration was allowed to occur in a truly three-dimensional solid representation of the brain. The elapsed time between diagnosis and death for this virtual glioma is approximately 158 days.

shows three perpendicular cross-sections (coronal, sagittal and horizontal or axial) of the virtual human brain intersecting in the white matter of the brain marked by an asterisk in the superior frontal region where the virtual tumour originates. The grey and white matters of the brain domain appear grey and white, respectively. A contour plot of the tumour cell density is represented in colour with red denoting a high density and blue denoting a low density. In each image, a single thick black curve defines the edge of the tumour that the model suggests would be detectable on enhanced CT associated with a threshold of detection of 8000 cells mm^−3^. The outermost light blue profile corresponds to an arbitrary threshold of detection 80 times more sensitive than enhanced CT (i.e. 100 cells mm^−3^). The left column of images in Figure 1 represents the tumour at the time of detection, defined as an enhanced CT-detectable tumour with average diameter of 3 cm, while the right column represents the tumour at the time of death, defined by an enhanced CT-detectable tumour with average diameter of 6 cm. The model predicts that there are approximately 158 days between diagnosis and death for a virtual patient with a glioblastoma defined by the given parameter estimates in this location in the frontal lobe.

Figure 2

**Figure 2:**
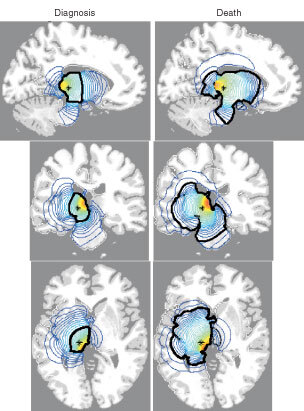
Sections of the virtual human brain in sagittal, coronal and horizontal planes that intersect at the site of the glioma originating in the thalamus denoted by an asterisk (*). The left column of brain sections corresponds to the tumour at diagnosis whereas the right column represents the same tumour at death. Red denotes a high density of tumour cells while blue denotes a low density. A thick black contour defines the edge of the tumour detectable by enhanced CT. Cell migration was allowed to occur in a truly three-dimensional solid representation of the brain. The elapsed time between diagnosis and death for this virtual gliomas is approximately 256 days.

shows a simulation similar to that presented in Figure 1 but the virtual tumour originates at a point (asterisk) in the deep grey matter of the thalamic region of the brain. These sites were chosen as two of the most commonly observed in patients in whom CT and MRI observations were correlated with histologic findings. The model predicts that there are approximately 256 days between diagnosis and death for a virtual patient with a glioblastoma defined by the given parameter estimates in this location in the thalamus.

Note the significant difference in survival times predicted for these two tumours. Previous two-dimensional models have suggested that tumour location can have a significant impact on survival time based simply on the local invasion abilities of the tumour (Swanson, 1999; Swanson *et al*, 2000).

Figure 3

**Figure 3:**
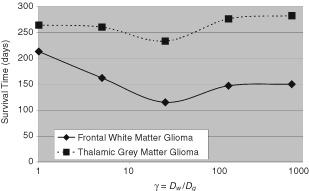
The effects of on survival times of five-fold variations of the ratio of motility in grey matter to that in white matter (γ=*D_w_*/*D_g_*) for virtual gliomas in frontal white matter or in thalamus.

shows the survival times (time between diagnosis at 3 cm diameter and death at 6 cm diameter) predicted by the model for a range of values of the ratio of the tumour cell migration rate in white matter to that in grey matter (γ= D_*w*_/D_*g*_). In order to compare across different virtual cases, the ratio γ is allowed vary such that the average diffusion coefficient over the entire brain is constant at 0.0013 cm^2^ day^−1^, the typical motility of a high grade glioma. To maintain this relationship we use the formula:

Average motility coefficient for entire brain = Motility coefficient in white matter × Volume fraction of brain that is white matter + Motility coefficient in grey matter × Volume fraction of brain that is grey matter

Or, equivalently,

0.0013 cm^2^ day^−1^=0.5723×*D_g_*+0.4277×*D_w_*

Recalling that *D_w_*=γ*D_g_*, then for each value of the ratio γ, we have

*D_g_*= 0.0013/(0.5723+0.4277 γ)

The volume fractions of grey and white matter were extracted from the BrainWeb database as 0.5723 and 0.4277, respectively (Collins *et al*, 1998).

In Figure 3, we note that the thalamic tumour consistently is predicted to take longer to become of fatal size. This relates to the relatively slow rate at which the tumour cells migrate in the deep grey matter in and around the thalamus whereas the frontal lesion would be expected to migrate γ-fold more quickly within the local white matter. There are no data to support or refute this difference in survival for thalamic tumours versus frontal white matter lesions. Due to the biological eloquence of the tissue within and in the region of the thalamus, one might expect these tumours to behave differently in terms of clinical symptoms resulting in perhaps a smaller size at diagnosis as well as at death. The simulation results we have tabulated here do not consider the degrees of eloquence of the brain tissue but simply track the progression of the growth of the lesion from a typical diagnostic size of 3 cm in diameter to a typical fatal size of 6 cm in diameter.

## DISCUSSION

What is abundantly clear from the figures is how far tumour cells have diffused beyond any current range of detection. Previous studies of the motility of gliomas have demonstrated that diffusion is an accurate estimation for the spread of gliomas (Woodward *et al*, 1996; Swanson, 1999). A consequence of modeling cellular motility by Fickian or gradient-driven diffusion, is the lack of a definitive interface between malignant and normal tissue. This mathematical consequence is correlated with the actual biology of human gliomas. Consider using CT or MRI images to delineate the possible interface between cancerous and normal tissue. Radical resection of the tumour even well beyond these interfaces has been shown to fail in numerous studies, as summarized by Nazzaro and Neuwelt (1990). Clearly, tumour cells invade peripheral to the CT- or MRI-defined boundaries of the tumour. Even standard histopathological analysis, one of our most sensitive means of detecting glioma cells, fails in locating all of the tumour cells, as was demonstrated by Silbergeld and Chicoine (1997).

Figures 1 and 2 show the spatio-temporal invasion of virtual gliomas at the time of diagnosis and death. These simulations clearly reveal the subthreshold invasion of the tumour well beyond the detectable portion of the tumour. No matter the extent of resection, the mathematical model indicates that the gross tumour will ultimately recur and kill.

### Resection and other localized treatment methods

Matsukado *et al*. (1961) analyzed 100 autopsy specimens of supratentorial glioblastomas searching for clues that might favour successful resection. They reported that 53 cases appeared grossly and microscopically confined to one cerebral hemisphere, 36 of these limited to the cerebral cortex and white matter and 17 invading the basal ganglia, internal capsule or diencephalon. Of the other cases, 25 crossed the midline, 15 of these also invading the contralateral basal ganglia and deep nuclei. They, of course, did not consider the hypothesis that drove the development of our mathematical models nearly 40 years later, namely that the two most important factors defining the pattern of glioblastomas are the growth rate and the diffusion coefficient. Considered in the present light, however, the patterns of their lobar and multilobar cases suggest that the major significant difference is the diffusion coefficient: low in those that remain relatively localized to the cortex and white matter (36 cases), higher in those that invade deep cerebral nuclei unilaterally (17 cases), still higher in those that cross the midline through the corpus callosum (10 cases) and highest in those that invade into the contralateral deep cerebral nuclei (15 cases). Matsukado *et al*. (1961) estimated that the 36 relatively localized cases could have been cured by radical surgery but Jelsma and Bucy (1967) failed to cure any even with extensive resection approaching hemispherectomy. This fits with our conclusion that submicroscopic invasion had taken place.

Although many attempts (radical resection and radiation therapy) have been made to suggest that a boundary exists, gliomas are typically diffuse with no clear boundary defining the interface between pathological and normal tissue. In this article, we demonstrate that this behaviour is well modelled by a diffusion-based motility process. Elsewhere we demonstrate this type of models use in also predicting *in vitro* behaviour of glioma cell invasion (Swanson, submitted).

Unlike real patients with real gliomas, virtual patients with virtual gliomas can be analyzed by allowing any particular factor to vary while keeping all the other determining factors constant. Such isolation techniques, of course, require a mathematical model that has sufficient complexity to contain a realistic number of variables. The recent availability of simulated MRIs, with proportions of grey and white matter accurately indicated, permitted the development of this model which is sufficiently complex to allow different diffusion rates in grey and white matter (e.g. a five-fold increase in diffusion or migration in white matter) as well as to prevent spread across sulci.

The model we have developed is a simple one focusing on two key elements, namely diffusion and growth. Other variables can and should be introduced into the model as their relative importance is discovered. These variables could range from implications regarding the interaction of normal brain tissue with malignant tissue to effectiveness of angiogenic vessels in providing nutrients to the malignant tissue. Elsewhere, aspects concerning the effect of tumour cell supopulations with different migration and proliferation rates have been considered by Tracqui *et al* (1995) and Swanson (1999).

Previous studies have shown how to determine estimates for the key parameters of our model from patient scans (Tracqui *et al*, 1995; Burgess *et al*, 1997; Swanson, 1999). With these estimates the present model can be predictive as to where the tumour is likely to grow in real time. Assuming that classical clinical neuroanatomy related to ‘eloquent’ sites applies to brain tumours as well as strokes, we need to know the concentration of tumour cells in any particular site necessary to produce symptoms and/or death. Also, assuming that most gliomas swell, showing mass effect as tumours, we will probably have to apply topological mathematical formulations allowing the virtual brain to accommodate the swellings and distortions visible in the real brain. Even without these, however, what seems clear from these theoretical studies of virtual gliomas is that current imaging techniques are woefully inadequate for definitive decisions as to what constitutes the optimal treatment for patients with gliomas.
